# Spasmodic Dysmenorrhœa

**Published:** 1873-11

**Authors:** Matthews Duncan


Case of Spasmodic Dysmenorrhœa. —*Dr. Matthews Duncan.* The following case illustrates very clearly the symptoms of the so-called mechanical dysmenorrhœa which, according to some authorities, is in almost every instance due to a flexion of the uterus. Whatever may be the frequency of flexion of the uterus, and it varies greatly with different practitioners, it is certain that this bent condition of the organ may sometimes cause painful and irregular menstruation; but it would appear, from the evidence of good and unprejudiced observers, that the part which a flexed uterus plays in the female economy has been greatly exaggerated.

The patient is a strong-looking, stout, ruddy-complexioned female, and with the exception of the complaint mentioned, enjoys, and always has enjoyed, good health. The dysmenorrhœa commenced when patient was sixteen years old, at which age menstruation began, and has continued without intermission ever since. The discharge is preceded generally by vomiting, and the pain accompanying it is of so severe a character that it necessitates her lying in bed for four or five days. The pain is referred by her to the hypogastric and lumbar regions, more especially the former.

One case proves very little, but this is a good example of the use and efficiency of treatment by bougies. The success certainly astonished the woman very much. This case also illustrates very distinctly one branch of the argument against the mere mechanical character of this dysmenorrhœa, which is more justly called spasmodic. It was a characteristic case of what is called mechanical dysmenorrhœa. The internal os uteri was very sensitive, tender, and rigid, yet it easily passed a No. 9 bougie, indicating a passage of dimensions quite natural, and more than sufficient to transmit the menstrual flow. Moreover, in this case the state of the internal os during the flow was examined, and it was then found enlarged, not contracted; it then allowed a No. 14 bougie to pass easily. In short, with all the symptoms of so-called mechanical dysmenorrhœa, there was not only no obstruction, but more than usual enlargement of the passage of exit for the menstrual fluid.

